# Obstructive sleep apnea as a modifier of endocrine toxicities associated with immune checkpoint inhibitors in lung cancer

**DOI:** 10.3389/fimmu.2026.1741875

**Published:** 2026-02-10

**Authors:** Lucrezia Pisanu, Pasquale Tondo, Francesco Bertuccio, Valentina Conio, Maria Arminio, Klodjana Mucaj, Elisabetta Gallo, Simone Montini, Jessica Saddi, Salvatore Corallo, Angelo G. Corsico, Giuseppe Insalaco, Maria Pia Foschino Barbaro, Giulia Scioscia, Francesco Fanfulla, Vito D’Agnano, Fabio Perrotta, Donato Lacedonia, Giulia M. Stella

**Affiliations:** 1 Unit of Respiratory Diseases, Department of Cardiothoracic and Vascular, IRCCS Policlinico San Matteo, Pavia, Italy; 2 Department of Medical and Surgical Sciences, University of Foggia, Foggia, Italy; 3 Respiratory and Intensive Care Unit, Department of Specialistic Medicine, Foggia Polyclinic University-Hospital, Foggia, Italy; 4 Department of Internal Medicine and Medical Therapeutics, University of Pavia Medical School, Pavia, Italy; 5 Unit of Radiation Therapy, Department of Onco-Hematology, IRCCS Policlinico San Matteo, Pavia, Italy; 6 Unit of Oncology, Department of Onco-Hematology, IRCCS Policlinico San Matteo, Pavia, Italy; 7 Institute of Translational Pharmacology, Italian National Research Council, Palermo, Italy; 8 Respiratory Function and Sleep Medicine Unit, IRCCS Maugeri Pavia and Montescano, Pavia, Italy; 9 Department of Translational Medical Sciences, University of Campania Luigi Vanvitelli, Naples, Italy; 10 Unit of Respiratory Medicine “Luigi Vanvitelli”, A.O. dei Colli, Monaldi Hospital, Naples, Italy

**Keywords:** endocrine toxicity, immunotherapy, lung cancer, metabolism, obstructive sleep apnea, ventilatory therapy

## Abstract

Obstructive sleep apnea (OSA) is one of the most common sleep disorders in the general population. It is characterized by recurrent alterations in nocturnal oxygenation, which have wide-ranging consequences on health. Beyond its well-established links to cardiovascular, neurocognitive, and metabolic diseases, recent evidence suggests a possible association between OSA and cancer, particularly lung cancer, one of the leading causes of death worldwide. The advent of immunotherapy has significantly improved outcomes for lung cancer patients in both early and advanced stages. However, immunotherapy is frequently associated with endocrine toxicities, which may overlap or interact with the metabolic alterations observed in OSA. This perspective aims to emphasize the clinical relevance of diagnosing and treating OSA in lung cancer patients undergoing immunotherapy, as proper management could help optimize both therapeutic efficacy and overall health.

## 1 Introduction

Obstructive sleep apnea (OSA) is a frequently diagnosed disorder characterized by recurrent episodes of partial or complete collapse of the upper airway during sleep. The mechanisms underlying upper airway collapse are complex and multifactorial, involving obesity, craniofacial abnormalities, altered upper airway muscle function, pharyngeal neuropathy, and rostral fluid shifts toward the neck. These phenomena are associated with cyclic fluctuations in blood oxygen saturation and recurrent arousals during the nocturnal period with an increased respiratory effort, all of which contribute to sympathetic nervous system activation, oxidative stress, and systemic inflammatory response (1).

The intermittent hypoxia and sleep fragmentation are linked to endothelial dysfunction, vascular remodeling, and an elevated risk of cardiovascular morbidity and mortality (2–4). Growing evidences suggests a bidirectional relationship between OSA and several comorbidities, including heart failure, stroke, and the metabolic syndrome or metabolic dysregulation like insulin resistance, type 2 diabetes, obesity-related inflammation (5, 6).

The development of neurological and cognitive impairments are increasingly recognized as consequences of OSA. The sleep disruption, hypoxemia, and oxidative stress contribute to structural and functional brain changes, accelerating cognitive decline and increasing the risk of neurodegenerative diseases such as Alzheimer’s and Parkinson’s (7).

In recent years, growing attention has been directed toward the potential relationship between OSA and cancer. Experimental evidence strongly suggests that intermittent hypoxia may promote tumor initiation and progression through the activation of hypoxia-inducible factors, modulation of immune surveillance, and increased cellular proliferation (1). Sleep fragmentation has also been proposed as a co-factor contributing to tumorigenesis by enhancing systemic inflammation and oxidative stress. Epidemiological data, however, remain heterogeneous. Large-scale population studies have shown that OSA is associated with an increased incidence of certain malignancies, although results vary according to cancer type. For example, in a nationwide database, OSA was linked to a higher incidence of lung, liver, and kidney cancer, as well as melanoma, whereas risks for colorectal and breast cancers appeared lower (8). More recent analyses confirm that the effect of OSA on cancer may differ depending on tumor site, suggesting that site-specific mechanisms are involved (9). Focusing on lung cancer (LC), one of the leading causes of cancer-related mortality worldwide, several studies have reported that nocturnal hypoxia is more prevalent in patients with lung cancer than in those with other malignancies, and that it is associated with increased rates of disease progression and reduced overall survival (10). Importantly, in this cohort, severe OSA and hypoxic burden were independent predictors of mortality, regardless of cancer stage or treatment modality. These findings strengthen the hypothesis that OSA may not only predispose to LC onset but also influence its aggressiveness and prognosis. Nevertheless, the causal nature of this relationship remains debated. While hypoxia is a plausible biological driver, confounding factors such as smoking and obesity complicate the interpretation of epidemiological data (11). This perspective aims to raise awareness of the link between OSA and lung cancer, with the goal of stimulating the collection of data and analyses that can further explore this correlation, utilizing clinical data (age, sex, endocrinological conditions, type and stage of lung neoplasia), bringing into practice what has been studied so far in the literature, especially in relation to oncological treatment and its consequences. To the best of our knowledge the link between OSA and endocrine ICI-related toxicity has never been investigated although a significant interplay and biological interconnection sustain the need of clarify the role of OSA as modifier of endocrine axis during ICI treatment. This point gains even more interest when taking under consideration the high rates of OSA diagnosis and the incidence of cancer patients undergoing ICI therapy. Within respect to the current state of the art, this perspective points out on one hand the mechanistic hints and shared pathways through OSA is implicated in inducing this outcome and on the other in suggesting actionable strategies (the role of ventilatory therapy) that are rarely evaluated when starting immunotherapy.

### 1.1 Lung cancer and OSA: what we know

Sleep disorders and nocturnal hypoxemia are extremely prevalent in the LC population (12) and seriously affect quality of life (13). Interestingly, the effect of OSA on cancer may differ depending on tumor site, suggesting that site-specific mechanisms are involved. Higher incidences are associated with LC, liver and kidney cancers and melanoma whereas colorectal and breast cancer patients rarely are affected by OSA (8, 14). Notably, the risk of LC is increased in female hypoxic COPD patients and concomitant sleep apnea (15). According to a recent analysis based on six cohort studies and over six million participants, patients with OSA have a higher risk of developing LC compared to the general population (16, 17) and overall deserve dedicated screening and/or early intervention programs (18). However, despite some epidemiological evidence, further data are required to clarify the mechanist interconnection between OSA and LC (16, 19–22). Recent genetic analyses conducted using the Mendelian randomization method did not find a clear causal relationship between OSA and LC, raising questions about the possibility that other confounding factors, such as smoking and obesity, might explain this association (23, 24). On the contrary, recent studies explored the role of biomarkers related to cancer growth and immune system evasion as key components of the link between OSA and lung cancer. The PD-1 and PD-L1 proteins, which facilitate immune system evasion by tumor cells, the midkine (MDK) and paraspeckle component-1 (PSPC1) proteins, which contribute to cellular aggressiveness and lymphangiogenesis, respectively, have been proposed as potential biomarkers. All these proteins have been found at elevated levels in patients with moderate-to-severe OSA and lung cancer, indicating a potential predisposition to tumor progression in these individuals. Moreover, sleep fragmentation has also been associated with immune and metabolic changes that may facilitate tumor progression. Experimental studies suggest that sleep fragmentation induces abnormal macrophage migration and an altered response to oxidative damage, factors that may contribute to cancer aggressiveness (25).

### 1.2 The role of intermittent hypoxia

**Figure 1:**
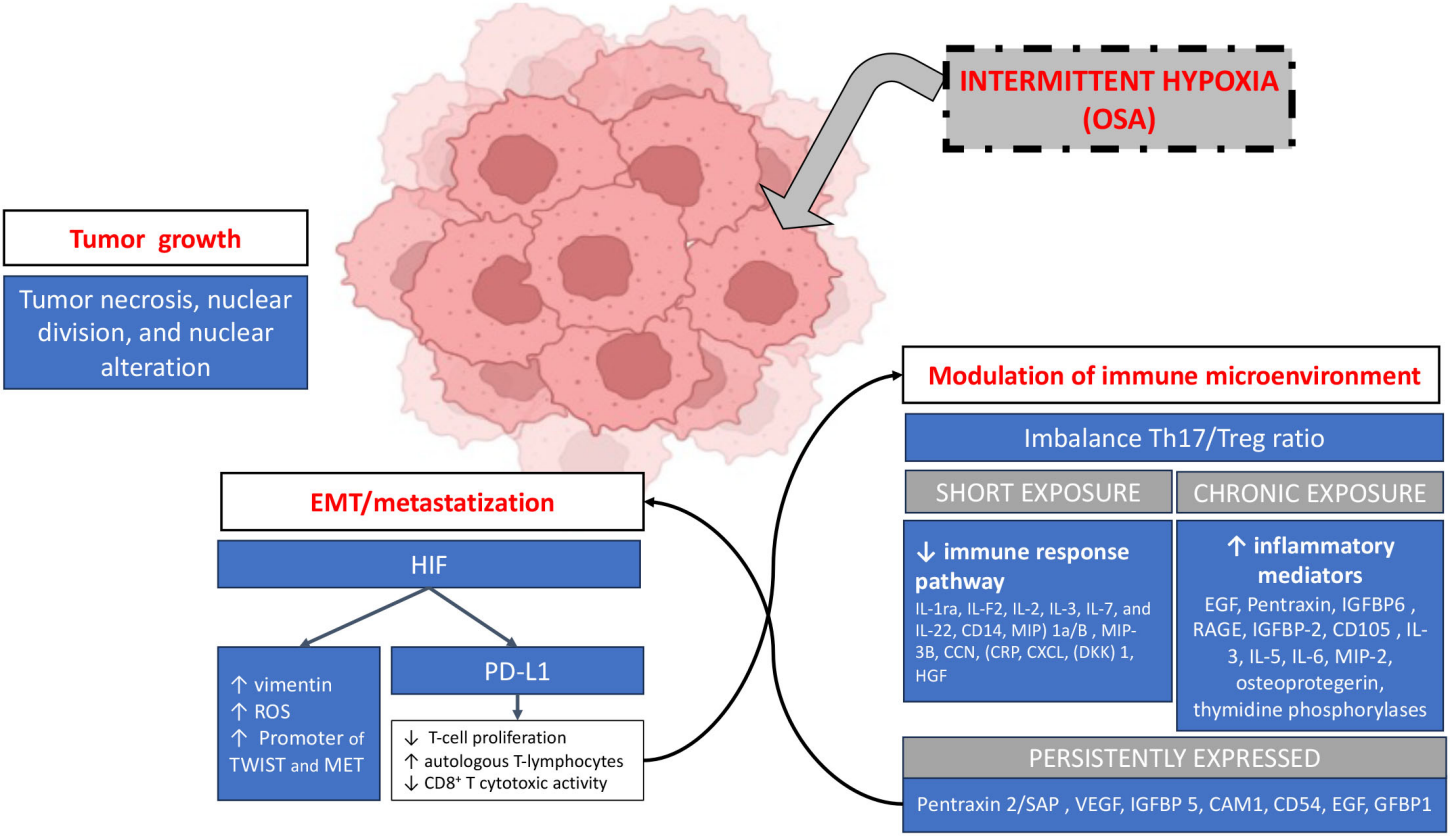
IH-mediated effects on LC and its microenvironment. IH acts by promoting cell alterations leading to tumor growth and it can also enhance tumor dissemination by increasing the expression of pro-invasive molecules and signals and by promoting tumor immune evasion. Moreover IH inferferes with tumor surrounding microenvironment mainly creating an imbalance in the Th17/Treg ratio, although some differences exist in experimental animal models between short exposure (reduced expression of immune-related molecules) to IH *vs* a chronic one (increased inflammatory expression); some pro-proliferative molecules are persistently expressed. Created in BioRender. ROS, reactive oxygen species; IL, interleukins; MP HGF, hepatocyte growth factor; MIP, Macrophage Inflammatory Protein-1; CCN, cellular Communication Networks factor; CRP, reactive protein C; CXCL, chemokine (C-X-C motif) ligand; DDK, DBF4-dependent kinase; IGF, insuline growth factor; RAGE, Receptor for Advanced Glycation Endproducts; IGFPB, Insulin-like Growth Factor Binding Protein; MIP, Major Intrinsic Protein; VEGF, vascular Endothelial Growth Farctor; EGF, Epithelial Growth Factor; HGF, hepatocyte growth factor; CAM, Cell Adhesion Molecule; SAP, Serum Amyloid P component; TWIST, gene encoding forn basic helix-loop-helix (bHLH) transcription factor; EMT, epithelial-to-mesenchymal transition.

To better understand the relationship between OSA and LC, it is essential to analyze the key mechanisms that promote cancer development and subsequent aberrant proliferation **Table 1**. Among the main biological markers involved in tumorigenesis are the vascular endothelial growth factor (VEGF), which plays a role in neoangiogenesis, the tumor growth factor (TGF-alpha 1), the tumor necrosis factor (TNF-alpha), the programmed cell death ligand (PD-L1), which enables immune system evasion by tumor cells, and certain genetic factors such as hypoxia-inducible genes (HIF-1alpha genes) are the main biological markers involved in tumorigenesis (26–28). As stated before, the intermittent hypoxia (IH) and sleep fragmentation are the most important physiological consequences of the episodes of apnea or hypopnea (29, 30). In LC, IH acts on all phases of tumor development, as detailed in **Figure 1**. Although a depper discussion of the role of intermittent hypoxia vs sustained hypoxia in tumorigenesis goes beyond the scope of this perspective, it should be underlined that - although not yet shown in case of pneumocytes - IH is known to induce cell lineage dysfunction and cell plasticity in several contexts (31–33). This behavior should be involved in alteration of cell differentiation and hierarchical organization that ultimately lead to cancer Moreover, IH in squamous cell cancer has been associated with the arousal of a more aggressive and undifferentiated phenotype thorough the expression of stemness-related markers as ALDHhi/EpCAM and the inhibition of differentiation molecules (involucrin) (34). Tumor progression should be blocked by restoring oxygenation and also by acting on modulation of IH-associated gene expression signatures. For instance, MiR-210-3p can favor IH-induced tumor progression by impairing E2F transcription factor 3 (E2F3) thus emerging a promising target in patients affected by OSA and cancer (35). IH activates hypoxia-inducible factor (HIF-1α), a protein that regulates the cellular response to hypoxia and is implicated in cancer growth and in vessels (neovascularization), essential elements for tumor development (36–38). Interestingly, intermittent hypoxia does not have the same role or effects on all tumor phenotypes. This variability may be related to the different sensitivity of cancer cells to hypoxic stimuli and their density of active VEGF receptors. The characteristics of hypoxia itself can also have different impacts on tumor cells: some studies have shown that lung squamous cell carcinoma cells tend to have a higher proliferation rate when exposed to intermittent hypoxia compared to sustained hypoxia (34, 39, 40). On the other hand, lung adenocarcinoma cell lines seem to respond better to prolonged hypoxia than to intermittent hypoxia (41). The mechanisms underlying these different responses are not yet fully understood (9). Additionally, sleep fragmentation appears to be associated with increased oxidative stress at the cellular level, a persistent inflammatory response, and neuroendocrine and immune dysfunctions, all of which are factors linked to an increased risk of tumorigenesis (42). However, the complexity and heterogeneity of OSA make it difficult to assess a clear causal relationship between OSA and LC, considering also the difficulty of accurately measuring the effects of intermittent hypoxia. Methodological limitations primarily stem from the observational nature of the studies, which exposes them to potential biases and confounding variables such as smoking, age, and obesity. Moreover, OSA is a heterogeneous condition that can manifest in various ways, with variable physiological impacts depending on severity and duration. Genetic studies, such as Mendelian randomization, have been useful in reducing observational biases, but the lack of a confirmed causal link indicates the need for further research to explore the role of OSA in the context of genetic-environmental interactions. Finally, most of the studies were predominantly focused on NSCLC, and its role in tumor progression, including metastasis. Moreover, if OSA were to be identified as an independent risk factor for lung cancer development, CPAP therapy could acquire a preventive role by targeting a modifiable mechanism such as intermittent hypoxia (43, 44). However, clinical evidence is still limited. Nonetheless, the assessment of the role of OSA in the natural history of LC may have positive implications since OSA is a modifiable risk factor: screening, diagnosis and treatment protocols have been standardized and available over the world (9, 45). However, further studies are needed to test this hypothesis, since clinical evidence is still limited. Justeau et al. in a large multicenter cohort of cancer-free OSA patients observed that the cancer incidence was associated with increasing severity of OSA. They found that, after adjustment for anthropometic data, smoking and alcohol consumption, comorbid cardiac, metabolic, and respiratory diseases, marital status, the level of sleep hypoxia (measured as % of sleep time spent with SpO2<90%) was associated with cancer incidence, particularly with lung and breast malignancies (15). However, the same research group in a subsequent 5-year follow-up study found that adherence to CPAP therapy was not associated with a reduction in all cancer incidence. However, there was a trend toward a significantly lower all-cancer incidence in CPAP adherent patients with more severe nocturnal hypoxaemia during the diagnostic test. In a large 30-year prospective cohort study performed in Scotland, it was demonstrated that long-term CPAP use reduces mortality, particularly that related to cancer (21).

**Table 1:** Biomarkers and risk factors connecting OSA and lung cancer.

Shared biomarker			
Biomarker	Role in OSA/lung cancer	Underlying biological mechanism	Evidence level
HIF-1α	Activated by IH (ntermittent hypoxia); central in tumor survival and progression.	Hypoxia-responsive transcription factor → angiogenesis, glycolysis, metabolic reprogramming.	Strong mechanistic rationale; preclinical and translational data.
VEGF	Up-regulated in IH (intermittent hypoxia); essential for tumor angiogenesis.	Promotes neovascularization, growth and metastatic potential.	Consistent across IH models and tumor phenotypes.
PD-1/PD-L1	Expressed in hypoxic/inflammatory milieu; facilitate immune evasion.	Inhibition of T-cell antitumor activity; higher levels in moderate–severe OSA and lung cancer.	Emerging evidence in clinical cohorts.
TGF-β/TGF-α1	Involved in proliferation, epithelial–mesenchymal transition (EMT), stromal remodeling.	TGF-β drives CAF (Cancer-Associated Fibroblasts) activation and invasion; OSA-IH potentiates TGF-β signaling.	Preclinical data: OSA/IH promotes invasion via TGF-β.
TNF-α	Elevated in OSA; contributes to tumorigenesis.	NF-κB activation, chronic inflammation, tumor cell survival.	Observational/translational evidence.
Midkine (MDK)	Linked to tumor aggressiveness; increased in OSA.	Cytokine promoting proliferation, migration, angiogenesis.	Reported in OSA+lung cancer patients.
PSPC1	Implicated in lymphangiogenesis and tumor dissemination; higher in OSA with lung cancer.	Transcriptional regulation of pro-metastatic genes.	Early experimental/clinical evidence.
Risk factors/potential correlations			
Factor/correlation		Evidence summary	Proposed mechanism
Presence and severity of OSA		Higher incidence of lung cancer in OSA patients in large cohorts; Mendelian randomization analyses failed to confirm direct causality, suggesting confounding factors (smoking, obesity, age).	Intermittent hypoxia (IH) → activation of HIF-1α → up-regulation of VEGF, angiogenesis, vascular remodeling, tumor growth.
Sleep fragmentation		Associated with oxidative stress, immune–metabolic dysfunction; experimental studies suggest enhanced tumor aggressiveness	↑ROS, chronic inflammation, impaired macrophage migration and immune surveillance → pro-tumorigenic microenvironment
Hypoxia pattern and tumor histology		*In vitro*: squamous cells proliferate more under IH vs sustained hypoxia; adenocarcinoma responds more to chronic hypoxia	Different hypoxic sensitivities; VEGF receptor density; metabolic plasticity across histotypes
Endocrine/metabolic alterations in OSA		May interact with endocrine toxicities from immune checkpoint inhibitors (ICI), complicating clinical outcomes	Insulin resistance, HPA axis activation, pro-inflammatory cytokines → crosstalk with immune pathways targeted by ICIs.
Confounding factors (smoking, obesity, age, male sex)		Likely contribute to OSA–lung cancer association; MR studies suggest no autonomous causal link of OSA	Independent carcinogenic effects (e.g., smoking); obesity as shared driver of both OSA and cancer
CPAP as a modifiable factor		Hypothesized preventive role by reducing IH; clinical evidence in lung cancer remains scarce	Correction of IH → down-regulation of HIF-1α/VEGF axis, improved immuno-metabolic homeostasis.

## 2 Perspective

### 2.1 Tumor immune checkpoints and inhibition therapy

Tumors express checkpoint proteins on their cell surfaces to escape detection by the immune system. Targeted inhibition of these receptors is the basis for the principle that T cell responses to tumors improve by blocking their progression. The last decade has seen the rapid development of immunotherapy as a viable therapeutic strategy against cancer. The introduction of immunotherapy into clinical practice has significantly changed the therapeutic strategy of patients with advanced NSCLC thanks to the important benefits offered in terms of long-term survival, safety and quality of life. A primary role in this field is played by so-called immune checkpoints, molecules on the surface of cells that can send inhibitory stimuli to attenuate immune responses. Tumors express “checkpoint” proteins on their surface to escape detection by the immune system. The CTLA-4 (Cytotoxic T-lymphocyte-associated Antigen-4) and PD-1 (Programmed cell Death protein-1) signaling pathways and its ligand PD-L1 are two of the many immune checkpoint pathways that play a critical role in controlling T cell immune responses against lung cancer. The CTL4 molecule is induced in T cells upon initial response to the antigen Inhibition of these receptors is the basis of immunotherapy, which promotes and enhances the T-cell response to the tumor (46–50). Moreover, it is widely demonstrated that targeted therapy and adjuvant immunotherapy can improve the outcomes of patients undergoing surgery selected on the basis of the expression of specific markers. The level of CTLA-4 expression depends on the extent of TCR-mediated signaling. Naive and memory T cells do not express CTLA-4 on their surface; after the TCR is activated by antigen encounter, CTLA-4 is transported to the cell surface. CTLA-4 functions as a signal attenuator to maintain a constant level of T cell activation. PD-1/PD-L1 play a major role in regulating inflammatory responses by effector T cells that recognize antigen in peripheral tissues. Activated T cells upregulate PD-1 and continue to express it in tissues. PD-L1 expression promotes immunosuppression. Immunosuppressive effects are linked to the induction of apoptosis of activated T cells, facilitation of anergy, and T cell exhaustion (51). Furthermore, PD-L1 expression is regulated by oncogenes and miRNAs (52, 53). Taken together, these immune checkpoint pathways are essential for maintaining peripheral immune tolerance and preventing excessive or autoreactive T-cell responses. By attenuating T-cell activation (CTLA-4) and limiting effector function in peripheral tissues (PD-1/PD-L1), they protect normal tissues from immune-mediated damage. Therefore, pharmacological blockade of these inhibitory signals with immune checkpoint inhibitors, while restoring antitumor immunity, also removes critical mechanisms of self-tolerance, predisposing patients to immune-mediated inflammatory reactions against healthy organs. Although IC inhibitors (ICIs) are generally better tolerated than common chemotherapy treatments (54), their mechanism of action results in a peculiar toxicity profile, characterized by immune-related adverse events (irAEs) that can potentially affect any organ or system and, although in most cases they are mild-moderate and reversible, in some cases they can be severe and/or fatal, especially if not promptly recognized and adequately treated. Given the ever-increasing diffusion of ICIs in the treatment of cancer patients, it is of fundamental importance that clinicians, patients themselves and their caregivers have adequate knowledge of the manifestations of ICI toxicity, for its early recognition and adequate treatment. Any organ or system can be affected by ICI toxicity. The most commonly affected organs are the skin, endocrine glands, colon, liver, and lung. The pattern, incidence, and severity of adverse events vary depending on the type of ICI (anti-CTLA-4 or anti-PD-1/PD-L1) and whether these drugs are used as single agents or in combination. Most irAEs occur within the first 3–4 months of starting treatment. The median time to onset of toxicity varies depending on the class of ICI used and the type of irAE. Late-onset irAEs have also been described in patients exposed to prolonged treatment with ICIs (55, 56). Overall, the widespread use of ICIs has made immune-related adverse events an important emerging clinical issue in oncology. Consequently, identifying specific patient conditions that may predispose or amplify immune-mediated toxicity is therefore becoming increasingly important. In this context, comorbidities that can alter immune regulation and tissue vulnerability, such as obstructive sleep apnea and associated endocrine and inflammatory dysregulation, may represent modifiers of both the efficacy and safety of immunotherapy.

### 2.2 OSA, immune modulation and ICI-related endocrine toxicity

The biologic and molecular interplay (synergic effects)? between the complex scenario characterized by endocrine disorders in cancer patients treated with immunotherapy and affected by OSA is still unknown. The incidence of OSA in unselected cancer population is varies from 5/10% to 30% and even higher if considering sleep disorders (57–61). The percentage of cancer patients who are eligible for ICI is about 30-40%, now increasing in consideration of combinatorial approaches and perioperative settings (62–65).

**Figure 2:**
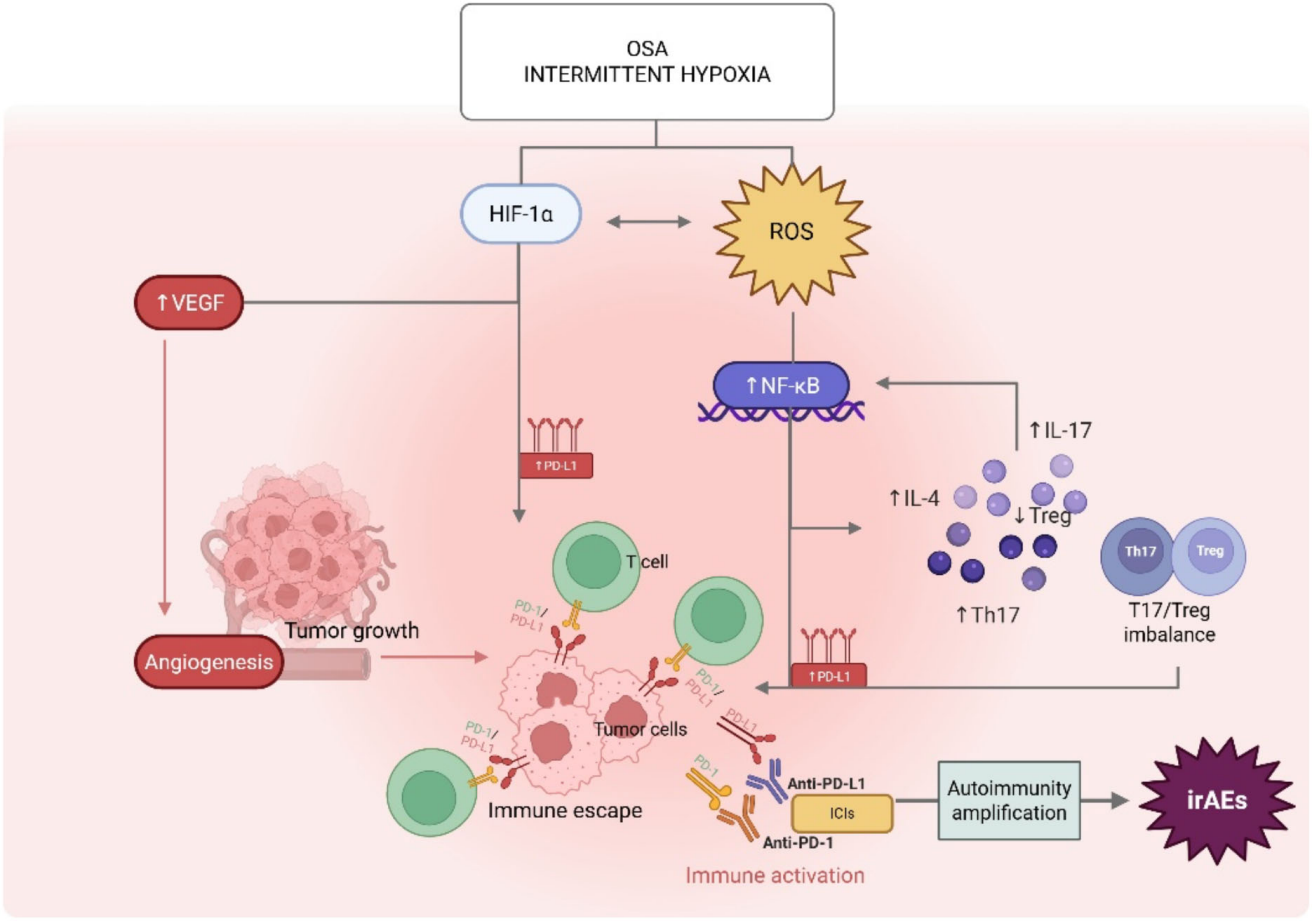
Proposed mechanistic link between OSA-related intermittent hypoxia and immune-related adverse events (irAEs) during ICI therapy. Intermittent hypoxia associated with obstructive sleep apnea (OSA) induces oxidative stress with increased generation of reactive oxygen species (ROS) and stabilization of hypoxia-inducible factor-1α (HIF-1α), which reciprocally amplify each other. HIF-1α up-regulates vascular endothelial growth factor (VEGF), promoting angiogenesis and tumor growth, and increases programmed death ligand-1 (PD-L1) expression on tumor and immune cells, facilitating immune escape. In parallel, ROS activate NF-κB signaling, leading to a pro-inflammatory cytokine milieu, increased Th17 responses, reduced regulatory T-cell (Treg) activity and an overall Th17/Treg imbalance (chronic inflammatory condition). This immune-dysregulated condition enhances PD-L1 expression and promotes tumor immune evasion. When immune checkpoint inhibitors (ICIs) targeting PD-1/PD-L1 are administered in this primed setting, removal of inhibitory signals results in amplified autoimmunity and a higher susceptibility to immune-related adverse events (irAEs). Created in BioRender. OSA, obstructive sleep apnea; ROS, reactive oxygen species; HIF-1α, hypoxia-inducible factor-1 alpha; VEGF, vascular endothelial growth factor; PD-1, programmed death-1; PD-L1, programmed death ligand-1; ICI, immune checkpoint inhibitor; NF-κB, nuclear factor k-B; Th17, T helper 17; Treg, regulatory T cell; irAEs, immune-related adverse events.

Experimental findings support the role of OSA in actively modulating PD-L1 expression and interfering with tumor immune surveillance. *In vivo* experiments have shown that OSA acts through IH as driver mechanism. Mice exposed to IH show increased PD-L1 expression in the tumor compared to normoxia-induced controls. This condition has been associated with upregulation of tumor associated macrophages (TAMs) in lung adenocarcinoma with concomitant OSA (40). Enhanced PD-L1 is also associated with increased tumor volume and weight in the IH model, suggesting that the hypoxic environment modifies the tumor microenvironment and immune evasion pathways (including PD-L1) in an OSA-like model. Moreover, in mice exposed to IH, the Th17/Treg ratio is increased, indicating that OSA can shift immunity toward a more pro-inflammatory and less regulatory state. Murine models of OSA display: i) changes in lung-specific inflammatory mediators (e.g., in the lung and other tissues) after chronic exposure to IH; ii) long-term increase in pro-inflammatory cytokines, which are part of the underlying immune activation that precedes and potentially modifies the response to ICIs (66–68). The observation of OSA-associated immune imbalance is of extreme relevance since this condition can predispose to autoimmunity or immune-mediated phenomena, as endocrine irAEs from ICIs. OSA-related IH can be considered an immune priming condition, implicated in the creation of a challenging microenvironment, activation of the NF-κB signaling cascade and NLRP3 inflammasome (69, 70), in which chronic inflammation and loss of immune tolerance create a biological substrate that can amplify the effects of immune checkpoint blockade. When ICIs are administered in this environment, the removal of inhibitory signals may result in an exaggerated immune response against normal endocrine tissues. Pharmacological block of PD-1/PD-L1 pair through ICI by removing inhibitory signals and promoting immune-inflammatory cascades, may trigger the onset autoimmune diseases which could be worsened in a IH context (**Figure 2**). The overall grade 3–4 ICI-related toxicities define severe, potentially life-threatening irAEs which require urgent intervention and most often hospitalization and immunosuppressive treatments. They occur in about 10-40% of cases, potentially involving many organs and systems and mainly the gastrointestinal tract (*e.g.* severe colitis, hepatitis), the lungs (pneumonitis and interstitial lung diseases), the heart (myocarditis), the neuromuscular disorder as myositis and myastenia, rheumatological toxicities, the skin (rash) and endocrine glands (hypophysitis, thyroiditis) (71–74).

**Figure 3:**
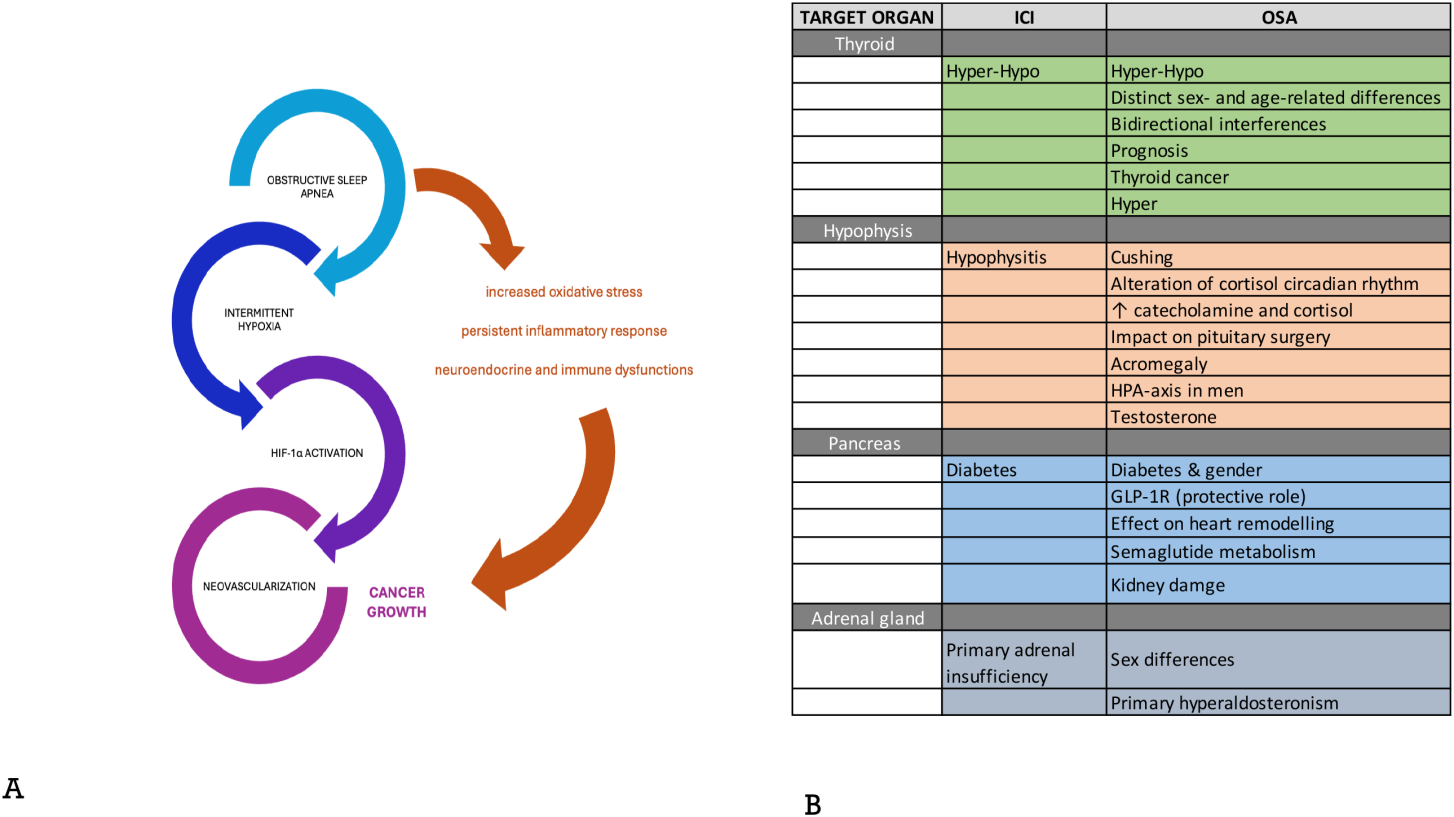
The complex mechanistic interconnection between OSA and Lung Cancer **(A)** and between OSA and ICI on endocrine glands **(B)**.

The endocrine events are among the most common, affecting up to 40% of treated patients (75, 76), and are described in international and national guidelines (74, 77, 78). Hormone-related side effects are quite common and might be permanent after ICI discontinuation (75) and those related to the thyroid, hypophysis, pancreas and adrenal gland are more frequent and better known. Sometimes, toxicity arises as painless inflammatory reaction as in thyroiditis or hypophysitis and consequent impairment of hormone homeostasis, but otherwise as secondary gland insufficiency or failure due to reduced pituitary secretion of adrenocorticotropic hormone (ACTH) or central hypothyroidism (79, 80). Although rare, endocrine damage could present as an acute event or even emergency as diabetic ketoacidosis (81). Compared with the general population, the prevalence of OSA in increased in endocrine and metabolic diseases, and, on the other hand OSA itself induces endocrine disorders (82, 83) (**Figure 3**). OSA is associated with thyroid damage (thyroiditis) and hormonal axis disruption (84–90) that can severely impair on patient’s prognosis (91). Distinct sex- and age-related different patterns are known (92–95). Thyroid malignant transformation has been described in association with OSA (96–99). Moreover, OSA has been associated with pituitary inflammation and hormones (100, 101) and axis alterations (102–106), leading to different conditions as Cushing (107), alteration of circadian rhythm (108), acromegaly (109). A large amount of literature has shown that diabetes is related to OSA, in some instances with sex and gender differences (110–119) and that OSA can interfere in hypoglycemic agents, also the novel ones (120). Secondary damage on kidney and heart has been reported (121, 122). As above discussed large amount of experimental data underline the high and early sensitivity to IH of many cellular elements of the nervous system (123–126); interestingly a sort of lung-brain axis in OSA has been also postulated (127). It is, thus conceivable that the most relevant endocrine axes deserving pritirization should be the hypothalamic-pituitary one. Sex- related differences have been frequently reported in modulating OSA effect on adrenal gland (128), which is mainly represented by primary hyperaldosteronism (129, 130). This interaction between OSA, immune dysregulation and endocrine vulnerability is particularly relevant in LC, where OSA is common and may contribute both to tumor progression and to an increased risk of severe immune-related toxicity during immunotherapy. Although the clinical implication of these observations is clearly evident, no clinical trial designed on OSA and ICI is currently available due to the lack of enough preclinical experiments and proper epidemiologic and demographic analysis, too. The translation of the proposed interaction into clinically actionable insights should be based on two different perspectives: i) the analysis of the effects of re-oxygenation in *in vitro* and *in vivo* cancer models; ii) validation of controversial effects of C-PAP as well as the evaluation of other therapeutical approaches (e.g. surgery) in more extensive cancer population. Notably, patient subgroups defined by sex, age, or lung cancer subtype, or of clinically meaningful outcomes such as severity, reversibility, or diagnostic delay should be considered in the design of clinical trial and in the results interpretation and validation.

## 3 Discussion

Current evidence indicates a clear association between OSA and LC. OSA is a relatively frequent comorbidity in LC patients and, through mechanisms such as intermittent hypoxia and immune dysregulation, may promote disease progression. Although definitive causal proof is still lacking, the incidence of LC in patients with OSA appears to be higher than in the general population, suggesting that OSA may represent a potential independent risk factor. In addition to influencing carcinogenesis, OSA can interfere with the therapeutic management of lung cancer, becoming particularly relevant in the context of immunotherapies. Indeed, the metabolic and endocrine alterations typical of OSA may add to or interact with the endocrine toxicities induced by immune checkpoint inhibition, amplifying their clinical impact. This dual interaction highlights the need to recognize and treat OSA in cancer patients in order not only to improve overall prognosis but also to prevent or mitigate severe toxicities related to immunotherapy.

## Data Availability

The original contributions presented in the study are included in the article/supplementary material. Further inquiries can be directed to the corresponding author.

## References

[1] Marrone O Bonsignore MR . Obstructive sleep apnea and cancer: a complex relationship. Curr Opin Pulm Med. (2020) 26:657–67. doi: 10.1097/MCP.0000000000000729, PMID: 32925366

[2] Riha RL . Defining obstructive sleep apnoea syndrome: a failure of semantic rules. Breathe (Sheff). (2021) 17:210082. doi: 10.1183/20734735.0082-2021, PMID: 35035552 PMC8753646

[3] Yeghiazarians Y Jneid H Tietjens JR Redline S Brown DL El-Sherif N . Obstructive sleep apnea and cardiovascular disease: A scientific statement from the american heart association. Circulation. (2021) 144:e56–67. doi: 10.1161/CIR.0000000000000988, PMID: 34148375

[4] Toffoli S Michiels C . Intermittent hypoxia is a key regulator of cancer cell and endothelial cell interplay in tumours. FEBS J. (2008) 275:2991–3002. doi: 10.1111/j.1742-4658.2008.06454.x, PMID: 18445039

[5] Lévy P Bonsignore MR Eckel J . Sleep, sleep-disordered breathing and metabolic consequences. Eur Respir J. (2009) 34:243–60. doi: 10.1183/09031936.00166808, PMID: 19567607

[6] Tondo P Scioscia G Sabato R Leccisotti R Hoxhallari A Sorangelo S . Mortality in obstructive sleep apnea syndrome (OSAS) and overlap syndrome (OS): The role of nocturnal hypoxemia and CPAP compliance. Sleep Med. (2023) 112:96–103. doi: 10.1016/j.sleep.2023.10.011, PMID: 37837825

[7] Ferini-Strambi L Lombardi GE Marelli S Galbiati A . Neurological deficits in obstructive sleep apnea. Curr Treat Options Neurol. (2017) 19:16. doi: 10.1007/s11940-017-0451-8, PMID: 28374233

[8] Gozal D Ham SA Mokhlesi B . Sleep apnea and cancer: analysis of a nationwide population sample. Sleep. (2016) 39:1493–500. doi: 10.5665/sleep.6004, PMID: 27166241 PMC4945307

[9] Gottlieb DJ Punjabi NM . Diagnosis and management of obstructive sleep apnea: A review. JAMA. (2020) 323:1389–400. doi: 10.1001/jama.2020.3514, PMID: 32286648

[10] Ferreira PM Carvalho I Redondo M van Zeller M Drummond M . The role of obstructive sleep apnea and nocturnal hypoxia as predictors of mortality in cancer patients. Sleep Med. (2024) 121:258–65. doi: 10.1016/j.sleep.2024.07.017, PMID: 39029304

[11] Huang HY Lin SW Chuang LP Wang CL Sun MH Li HY . Severe OSA associated with higher risk of mortality in stage III and IV lung cancer. J Clin Sleep Med. (2020) 16:1091–8. doi: 10.5664/jcsm.8432, PMID: 32209219 PMC7954049

[12] Cabezas E Pérez-Warnisher MT Troncoso MF Gómez T Melchor R Pinillos EJ . Sleep disordered breathing is highly prevalent in patients with lung cancer: results of the sleep apnea in lung cancer study. Respiration. (2019) 97:119–24. doi: 10.1159/000492273, PMID: 30261487

[13] Zidan MH Shaarawy HM Gharraf HS Helal SF Hassan M Rizk R . Predictors of moderate to severe obstructive sleep apnea in patients with lung cancer. Respir Res. (2024) 25:197. doi: 10.1186/s12931-024-02789-z, PMID: 38715026 PMC11077845

[14] Soccio P Moriondo G Scioscia G Tondo P Bruno G Giordano G . MiRNA expression affects survival in patients with obstructive sleep apnea and metastatic colorectal cancer. Noncoding RNA Res. (2024) 10:91–7. doi: 10.1016/j.ncrna.2024.09.010, PMID: 39315340 PMC11419774

[15] Proesmans K Luik AI Lahousse L . Sex-specific associations between sleep apnoea and lung cancer risk in patients with COPD: a nationwide prospective cohort study. Lancet Reg Health Eur. (2025) 52:101269. doi: 10.1016/j.lanepe.2025.101269, PMID: 40224371 PMC11987683

[16] Martínez-García MÁ Oscullo G Gómez-Olivas JD Inglés-Azorin M Mompeán S Proesmans K . Is obstructive sleep apnea a risk factor for lung cancer? - from pathophysiological mechanisms to clinical data. Ann Trans Med. (2023) 11:422. doi: 10.21037/atm-23-1641, PMID: 38213801 PMC10777209

[17] Cui Z Ruan Z Li M Ren R Ma Y Zeng J . Obstructive sleep apnea promotes the progression of lung cancer by modulating cancer cell invasion and cancer-associated fibroblast activation via TGFβ signaling. Redox report: Commun Free Radical Res. (2023) 28:2279813. doi: 10.1080/13510002.2023.2279813, PMID: 38010093 PMC11001276

[18] Al-Nusair M Obeidat O Alagha Z Wright T Al-Momani Z Alnabahneh N . Mahdi A Unmasking the link between sleep apnea and lung cancer risk: A retrospective propensity-matched cohort study. JCO. (2025) 43:10558–8. doi: 10.1200/JCO.2025.43.16_suppl.10558

[19] Justeau G Gervès-Pinquié C Le Vaillant M Trzepizur W Meslier N Goupil F . Association between nocturnal hypoxemia and cancer incidence in patients investigated for OSA: data from a large multicenter french cohort. Chest. (2020) 158:2610–20. doi: 10.1016/j.chest.2020.06.055, PMID: 32629036

[20] Yuan F Hu Y Xu F Feng X . A review of obstructive sleep apnea and lung cancer: epidemiology, pathogenesis, and therapeutic options. Front Immunol. (2024) 15:1374236. doi: 10.3389/fimmu.2024.1374236, PMID: 38605948 PMC11007033

[21] Martinez-Garcia MA . Linking obstructive sleep apnoea and lung cancer: a further step down the road. ERJ Open Res. (2024) 10:01050–2023. doi: 10.1183/23120541.01050-2023, PMID: 38375432 PMC10875466

[22] Cho J Jo S . Association of obstructive sleep apnea with risk of lung cancer: a nationwide cohort study in Korea. Sci Rep. (2024) 14:12394. doi: 10.1038/s41598-024-63238-x, PMID: 38811831 PMC11137051

[23] Chen J Liu W Li P Liu Y Wang Z Liu H . Causal effect of potential risk factors on obstructive sleep apnea: a Mendelian randomization study. Future Sci OA. (2025) 11:2541530. doi: 10.1080/20565623.2025.2541530, PMID: 40752015 PMC12320811

[24] Yao J Duan R Li Q Mo R Zheng P Feng T . Association between obstructive sleep apnea and risk of lung cancer: findings from a collection of cohort studies and Mendelian randomization analysis. Front Oncol. (2024) 14:1346809. doi: 10.3389/fonc.2024.1346809, PMID: 39070143 PMC11272613

[25] Dodds S Williams LJ Roguski A Vennelle M Douglas NJ Kotoulas SC . Mortality and morbidity in obstructive sleep apnoea-hypopnoea syndrome: results from a 30-year prospective cohort study. ERJ Open Res. (2020) 6:00057–2020. doi: 10.1183/23120541.00057-2020, PMID: 32963994 PMC7487348

[26] Stella GM Luisetti M Pozzi E Comoglio PM . Oncogenes in non-small-cell lung cancer: emerging connections and novel therapeutic dynamics. Lancet Respir Med. (2013) 1:251–61. doi: 10.1016/S2213-2600(13)70009-2, PMID: 24429131

[27] Lacedonia D Scioscia G Pia Palladino G Gallo C Carpagnano GE Sabato R . MicroRNA expression profile during different conditions of hypoxia. Oncotarget. (2018) 9:35114–22. doi: 10.18632/oncotarget.26210, PMID: 30416683 PMC6205556

[28] Hanahan D Weinberg RA . Hallmarks of cancer: the next generation. Cell. (2011) 144:646–74. doi: 10.1016/j.cell.2011.02.013, PMID: 21376230

[29] Almendros I Gozal D . Intermittent hypoxia and cancer: Undesirable bed partners? Respir Physiol Neurobiol. (2018) 256:79–86. doi: 10.1016/j.resp.2017.08.008, PMID: 28818483

[30] Bonsignore M Almendros I Bouloukaki I Schiza S . Chapter 7 - Interconnections between nocturnal respiratory disorders and cancer. In: Mogavero MP Lanza G Ferini-Strambi L Ferri R , editors. Sleep and Cancer. Academic Press (2026), ISBN: ISBN 9780443289385. p. 117–78. doi: 10.1016/B978-0-443-28938-5.00014-3

[31] Xie Y Wu S Sun YY Chen H Ding X Pei G . Chronic intermittent hypoxia causes oligodendrocyte lineage cell dysfunction and cognitive deficits in a murine model: Partial reversal by reoxygenation treatment. Sleep Med. (2025) 136:106864. doi: 10.1016/j.sleep.2025.106864, PMID: 41106043

[32] Khuu MA Pagan CM Nallamothu T Hevner RF Hodge RD Ramirez JM . Intermittent hypoxia disrupts adult neurogenesis and synaptic plasticity in the dentate gyrus. J Neurosci. (2019) 39:1320–31. doi: 10.1523/JNEUROSCI.1359-18.2018, PMID: 30587544 PMC6381238

[33] Liu Q Palmgren VAC Danen EH Le Dévédec SE . Acute vs. chronic vs. intermittent hypoxia in breast Cancer: a review on its application in *in vitro* research. Mol Biol Rep. (2022) 49:10961–73. doi: 10.1007/s11033-022-07802-6, PMID: 36057753 PMC9618509

[34] Marhuenda E Campillo N Gabasa M Martínez-García MA Campos-Rodríguez F Gozal D . Effects of sustained and intermittent hypoxia on human lung cancer cells. Am J Respir Cell Mol Biol. (2019) 61:540–4. doi: 10.1165/rcmb.2018-0412LE, PMID: 31573339

[35] Zhang XB Song Y Lai YT Qiu SZ Hu AK Li DX . MiR-210-3p enhances intermittent hypoxia-induced tumor progression via inhibition of E2F3. Sleep Breath. (2024) 28:607–17. doi: 10.1007/s11325-023-02925-x, PMID: 37775619

[36] Moriondo G Soccio P Minoves M Scioscia G Tondo P Foschino Barbaro MP . Intermittent hypoxia mediates cancer development and progression through HIF-1 and miRNA regulation. Arch Bronconeumol. (2023) 59:629–37. doi: 10.1016/j.arbres.2023.07.001, PMID: 37517933

[37] Paradis A Lemarié E Pépin JL Godin-Ribuot D Briançon-Marjollet A . Differential impact of intermittent vs. Sustained hypoxia on HIF-1, VEGF and proliferation of HepG2 cells. Int J Mol Sci. (2023) 24:6875. doi: 10.3390/ijms24086875, PMID: 37108039 PMC10139223

[38] Stella GM Benvenuti S Comoglio PM . Targeting the MET oncogene in cancer and metastases. Expert Opin Investig Drugs. (2010) 19:1381–94. doi: 10.1517/13543784.2010.522988, PMID: 20868306

[39] Hao S Zhu X Liu Z Wu X Li S Jiang P . Chronic intermittent hypoxia promoted lung cancer stem cell-like properties via enhancing Bach1 expression. Respir Res. (2021) 22:58. doi: 10.1186/s12931-021-01655-6, PMID: 33596919 PMC7890965

[40] Guo X Liu Y Kim JL Kim EY Kim EQ Jansen A . Effect of cyclical intermittent hypoxia on Ad5CMVCre induced solitary lung cancer progression and spontaneous metastases in the KrasG12D+; p53fl/fl; myristolated p110fl/fl ROSA-gfp mouse. PLoS One. (2019) 14:e0212930. doi: 10.1371/journal.pone.0212930, PMID: 30811514 PMC6392281

[41] Cui Z Ruan Z Li M Ren R Ma Y Zeng J . Intermittent hypoxia inhibits anti-tumor immune response via regulating PD-L1 expression in lung cancer cells and tumor-associated macrophages. Int Immunopharmacol. (2023) 122:110652. doi: 10.1016/j.intimp.2023.110652, PMID: 37478668

[42] Davinelli S Medoro A Savino R Scapagnini G . Sleep and oxidative stress: current perspectives on the role of NRF2. Cell Mol Neurobiol. (2024) 44:52. doi: 10.1007/s10571-024-01487-0, PMID: 38916679 PMC11199221

[43] Fanfulla F Barbi V Bachetti T Maestri R Robbi E Mongelli A . Modulation of hypoxia-sensitive non-coding RNAs following continuous positive airway pressure therapy in obstructive sleep apnea in peripheral blood. Eur J Intern Med. (2025) 141:106393. doi: 10.1016/j.ejim.2025.06.022, PMID: 40603222

[44] Lo Bue A Salvaggio A Iacono Isidoro S Romano S Insalaco G . OSA and CPAP therapy: effect of gender, somnolence, and treatment adherence on health-related quality of life. Sleep Breath. (2020) 24:533–40. doi: 10.1007/s11325-019-01895-3, PMID: 31309464

[45] Benjafield AV Pepin JL Cistulli PA Wimms A Lavergne F Sert Kuniyoshi FH . Positive airway pressure therapy and all-cause and cardiovascular mortality in people with obstructive sleep apnoea: a systematic review and meta-analysis of randomised controlled trials and confounder-adjusted, non-randomised controlled studies. Lancet Respir Med. (2025) 13:403–13. doi: 10.1016/S2213-2600(25)00002-5, PMID: 40118084 PMC12045716

[46] Rabinovich GA Gabrilovich D Sotomayor EM . Immunosuppressive strategies that are mediated by tumor cells. Annu Rev Immunol. (2007) 25:267–96. doi: 10.1146/annurev.immunol.25.022106.141609, PMID: 17134371 PMC2895922

[47] Wellenstein MD de Visser KE . Cancer-cell-intrinsic mechanisms shaping the tumor immune landscape. Immunity. (2018) 48:399–416. doi: 10.1016/j.immuni.2018.03.004, PMID: 29562192

[48] Paulsen EE Kilvaer TK Rakaee M Richardsen E Hald SM Andersen S . CTLA-4 expression in the non-small cell lung cancer patient tumor microenvironment: diverging prognostic impact in primary tumors and lymph node metastases. Cancer Immunol Immunother. (2017) 66:1449–61. doi: 10.1007/s00262-017-2039-2, PMID: 28707078 PMC5645427

[49] Zhang H Dai Z Wu W Wang Z Zhang N Zhang L . Regulatory mechanisms of immune checkpoints PD-L1 and CTLA-4 in cancer. J Exp Clin Cancer Res. (2021) 40:184. doi: 10.1186/s13046-021-01987-7, PMID: 34088360 PMC8178863

[50] Shen S Hong Y Huang J Qu X Sooranna SR Lu S . Targeting PD-1/PD-L1 in tumor immunotherapy: Mechanisms and interactions with host growth regulatory pathways. Cytokine Growth Factor Rev. (2024) 79:16–28. doi: 10.1016/j.cytogfr.2024.08.001, PMID: 39179486

[51] Vitale M Pagliaro R Viscardi G Pastore L Castaldo G Perrotta F . Unraveling resistance in lung cancer immunotherapy: clinical milestones, mechanistic insights, and future strategies. Int J Mol Sci. (2025) 26:9244. doi: 10.3390/ijms26189244, PMID: 41009806 PMC12470719

[52] Santini FC Hellmann MD . PD-1/PD-L1 axis in lung cancer. Cancer J. (2018) 24:15–9. doi: 10.1097/PPO.0000000000000300, PMID: 29360723 PMC5784856

[53] Buchbinder EI Desai A . CTLA-4 and PD-1 pathways: similarities, differences, and implications of their inhibition. Am J Clin Oncol. (2016) 39:98–106. doi: 10.1097/COC.0000000000000239, PMID: 26558876 PMC4892769

[54] Santana-Davila R Chow LQ . The use of combination immunotherapies as front-line therapy for non-small-cell lung cancer. Future Oncol. (2018) 14:191–4. doi: 10.2217/fon-2017-0124, PMID: 29334785

[55] Durbin SM Zubiri L Perlman K Wu CY Lim T Grealish K . Late-onset immune-related adverse events after immune checkpoint inhibitor therapy. JAMA Netw Open. (2025) 8:e252668. doi: 10.1001/jamanetworkopen.2025.2668, PMID: 40146104 PMC11950896

[56] Lenti MV Ribaldone DG Borrelli de Andreis F Vernero M Barberio B De Ruvo M . A 1-year follow-up study on checkpoint inhibitor-induced colitis: results from a European consortium. ESMO Open. (2024) 9:103632. doi: 10.1016/j.esmoop.2024.103632, PMID: 38970840 PMC11360400

[57] Kendzerska T Leung RS Hawker G Tomlinson G Gershon AS . Obstructive sleep apnea and the prevalence and incidence of cancer. CMAJ. (2014) 186:985–92. doi: 10.1503/cmaj.140238, PMID: 25096668 PMC4162778

[58] Cao Y Ning P Li Q Wu S . Cancer and obstructive sleep apnea: An updated meta-analysis. Med (Baltimore). (2022) 101:e28930. doi: 10.1097/MD.0000000000028930, PMID: 35451384 PMC8913079

[59] Cheong AJY Tan BKJ Teo YH Tan NKW Yap DWT Sia CH . Obstructive sleep apnea and lung cancer: A systematic review and meta-analysis. Ann Am Thorac Soc. (2022) 19:469–75. doi: 10.1513/AnnalsATS.202108-960OC, PMID: 34792438

[60] Palm A Theorell-Haglöw J Isakson J Ljunggren M Sundh J Ekström MP . Association between obstructive sleep apnoea and cancer: a cross-sectional, population-based study of the DISCOVERY cohort. BMJ Open. (2023) 13:e064501. doi: 10.1136/bmjopen-2022-064501, PMID: 36868588 PMC9990651

[61] Büttner-Teleagă A Kim YT Osel T Richter K . Sleep disorders in cancer-A systematic review. Int J Environ Res Public Health. (2021) 18:11696. doi: 10.3390/ijerph182111696, PMID: 34770209 PMC8583058

[62] Raphael J Richard L Lam M Blanchette PS Leighl NB Rodrigues G . Utilization of immunotherapy in patients with cancer treated in routine care settings: A population-based study using health administrative data. Oncologist. (2022) 27:675–84. doi: 10.1093/oncolo/oyac085, PMID: 35552444 PMC9355820

[63] Haslam A Prasad V . Estimation of the percentage of US patients with cancer who are eligible for and respond to checkpoint inhibitor immunotherapy drugs. JAMA Netw Open. (2019) 2:e192535. doi: 10.1001/jamanetworkopen.2019.2535, PMID: 31050774 PMC6503493

[64] Desai A Schwed K Kalesinskas L Yuan Q Bryan J Keane C . Clinical outcomes of perioperative immunotherapy in resectable non-small cell lung cancer. JAMA Netw Open. (2025) 8:e2517953. doi: 10.1001/jamanetworkopen.2025.17953, PMID: 40587130 PMC12210081

[65] Semaan K Nawfal R Nally E Janjigian YY Robert C Peters S . Landscape of subsequent therapies in perioperative immunotherapy trials across multiple cancer types. Lancet Oncol. (2025) 26:161–4. doi: 10.1016/S1470-2045(24)00513-8, PMID: 39510116

[66] Huang MH Zhang XB Wang HL Li LX Zeng YM Wang M . Intermittent hypoxia enhances the tumor programmed death ligand 1 expression in a mouse model of sleep apnea. Ann Transl Med. (2019) 7:97. doi: 10.21037/atm.2019.01.44, PMID: 31019947 PMC6462651

[67] Park D-Y Kim C-H Park D-Y Kim HJ Cho H-J . Intermittent hypoxia induces Th17/Treg imbalance in a murine model of obstructive sleep apnea. PLoS One. (2024) 19:e0305230. doi: 10.1371/journal.pone.0305230, PMID: 38913648 PMC11195984

[68] Koritala BSC Gaspar LS Bhadri SS Massie KS Lee YY Paulose J . Murine pro-inflammatory responses to acute and sustained intermittent hypoxia: implications for obstructive sleep apnea research. Laryngoscope. (2024) 134 Suppl 4:S1–S11. doi: 10.1002/lary.30915, PMID: 37540033 PMC10838350

[69] Dong N Yue H . Advances in immunology of obstructive sleep apnea: mechanistic insights, clinical impact, and therapeutic perspectives. Front Immunol. (2025) 16:1654450. doi: 10.3389/fimmu.2025.1654450, PMID: 41194928 PMC12582966

[70] Yangzhong X Hua S Wen Y Bi X Li M Zheng Y . NLRP3 inflammasome triggers inflammation of obstructive sleep apnea. Curr Mol Med. (2025) 25:522–36. doi: 10.2174/0115665240294605240426123650, PMID: 38757326

[71] Kennedy LB Salama AKS . A review of cancer immunotherapy toxicity. CA Cancer J Clin. (2020) 70:86–104. doi: 10.3322/caac.21596, PMID: 31944278

[72] Kong X Chen L Su Z Sullivan RJ Blum SM Qi Z . Toxicities associated with immune checkpoint inhibitors: a systematic study. Int J Surg. (2023) 109:1753–68. doi: 10.1097/JS9.0000000000000368, PMID: 37132038 PMC10389211

[73] Chhabra N Kennedy J . A review of cancer immunotherapy toxicity: immune checkpoint inhibitors. J Med Toxicol. (2021) 17:411–24. doi: 10.1007/s13181-021-00833-8, PMID: 33826117 PMC8455777

[74] Haanen J Obeid M Spain L Carbonnel F Wang Y Robert C . Management of toxicities from immunotherapy: ESMO Clinical Practice Guideline for diagnosis, treatment and follow-up. Ann Oncol. (2022) 33:1217–38. doi: 10.1016/j.annonc.2022.10.001, PMID: 36270461

[75] Wright JJ Powers AC Johnson DB . Endocrine toxicities of immune checkpoint inhibitors. Nat Rev Endocrinol. (2021) 17:389–99. doi: 10.1038/s41574-021-00484-3, PMID: 33875857 PMC8769055

[76] Martins F Sofiya L Sykiotis GP Lamine F Maillard M Fraga M . Adverse effects of immune-checkpoint inhibitors: epidemiology, management and surveillance. Nat Rev Clin Oncol. (2019) 16:563–80. doi: 10.1038/s41571-019-0218-0, PMID: 31092901

[77] Associazione Italiana Oncologia Medica (AIOM) . Linee guida GESTIONE DELLA TOSSICITÀ DA IMMUNOTERAPIA. Available online at: https://www.iss.it/documents/20126/8403839/LG+200_Tox+da+immunoterapia_ed2023.pdf/703bb77e-5567-6675-8168-f3d0cddd1e4c?t=1696845726727 (Accessed January 07, 2026).

[78] Thompson JA Schneider BJ Brahmer J Zaid MA Achufusi A Armand P . NCCN guidelines^®^ Insights: management of immunotherapy-related toxicities, version 2.2024. J Natl Compr Canc Netw. (2024) 22:582–92. doi: 10.6004/jnccn.2024.0057, PMID: 39536465

[79] Cheng NH Lee H Balchander D Mimms R Krishnamurthy M . Immunotherapy induced adrenal insufficiency: an underdiagnosed cause of persistent hypotension in cancer. J Community Hosp Intern Med Perspect. (2024) 14:68–70. doi: 10.55729/2000-9666.1375, PMID: 39391114 PMC11464065

[80] Kristan MM Toro-Tobon D Francis N Desale S Bikas A Jonklaas J . Immunotherapy-associated hypothyroidism: comparison of the pre-existing with de-novo hypothyroidism. Front Endocrinol (Lausanne). (2022) 13:798253. doi: 10.3389/fendo.2022.798253, PMID: 35360059 PMC8962946

[81] Keerty D Das M Hallanger-Johnson J Haynes E . Diabetic ketoacidosis: an adverse reaction to immunotherapy. Cureus. (2020) 12:e10632. doi: 10.7759/cureus.10632, PMID: 33123445 PMC7584305

[82] Zhang H Wu Z Chen Q Yu G Chen L Ma Y . Subtype-specific causal effects of hypothyroidism on obstructive sleep apnea: A bidirectional Mendelian randomization study. Med (Baltimore). (2025) 104:e43266. doi: 10.1097/MD.0000000000043266, PMID: 40629611 PMC12237354

[83] Akset M Poppe KG Kleynen P Bold I Bruyneel M . Endocrine disorders in obstructive sleep apnoea syndrome: A bidirectional relationship. Clin Endocrinol (Oxf). (2023) 98:3–13. doi: 10.1111/cen.14685, PMID: 35182448

[84] Ghantasala M John NA Taranikanti M Kamble P Umesh M Gaur A . Polysomnographic evaluation of sleep quality and quantitative variables in patients of hypothyroidism. J Family Med Prim Care. (2025) 14:2716–20. doi: 10.4103/jfmpc.jfmpc_1872_24, PMID: 40814471 PMC12349815

[85] Zhai L Gao X . Recent advances in the study of the correlation between obstructive sleep apnea and thyroid-disorders. Sleep Breath. (2025) 29:176. doi: 10.1007/s11325-025-03350-y, PMID: 40323542

[86] Acharya H Jayaraj Mangala S Kalra P . Evaluating the spectrum of sleep abnormalities in patients with primary hypothyroidism. Cureus. (2024) 16:e69855. doi: 10.7759/cureus.69855, PMID: 39435197 PMC11493210

[87] Kumari N Arora N Das S Tiple S Singh H Patidar N . Assessment of risk of obstructive sleep apnea with thyroid eye disease and its activity. Indian J Ophthalmol. (2023) 71:3711–4. doi: 10.4103/IJO.IJO_912_23, PMID: 37991309 PMC10788756

[88] Pancholi C Chaudhary SC Gupta KK Sawlani KK Verma SK Singh A . Obstructive sleep apnea in hypothyroidism. Ann Afr Med. (2022) 21:403–9. doi: 10.4103/aam.aam_134_2, PMID: 36412342 PMC9850883

[89] Shi Y Cao Z Xie Y Yuan Y Chen X Su Y . Association between obstructive sleep apnea and thyroid function: A 10-year retrospective study. Sleep Med. (2023) 103:106–15. doi: 10.1016/j.sleep.2023.01.027, PMID: 36774744

[90] Masarwy R Kampel L Ungar OJ Warshavsky A Horowitz G Rosenzweig E . The impact of thyroidectomy on obstructive sleep apnea: a systematic review and meta-analysis. Eur Arch Otorhinolaryngol. (2022) 279:5801–11. doi: 10.1007/s00405-022-07461-0, PMID: 35723730

[91] Zhou Y He Q Ai H Zhao X Chen X Li S . The long-term prognostic implications of free triiodothyronine to free thyroxine ratio in patients with obstructive sleep apnea and acute coronary syndrome. Front Endocrinol (Lausanne). (2024) 15:1451645. doi: 10.3389/fendo.2024.1451645, PMID: 39351531 PMC11439673

[92] Xie Y Zhang H Cao Z Zhou Y Lu C Yin L . Associations among obstructive sleep apnea, thyroid function and morphology changes. Nat Sci Sleep. (2025) 17:1727–41. doi: 10.2147/NSS.S507318, PMID: 40756739 PMC12317735

[93] Zhou Y Shi Y Zhu S Cao Z Xie Y Lu C . Independent association of sleep apnea-specific hypoxic burden and sleep breathing impairment index with thyroid function in obstructive sleep apnea: A retrospective study. Nat Sci Sleep. (2025) 17:1543–56. doi: 10.2147/NSS.S525750, PMID: 40635995 PMC12239915

[94] Wang L Fang X Xu C Pan N Wang Y Xue T . Epworth sleepiness scale is associated with hypothyroidism in male patients with obstructive sleep apnea. Front Endocrinol (Lausanne). (2022) 13:1010646. doi: 10.3389/fendo.2022.1010646, PMID: 36465644 PMC9708720

[95] Fernandes GL Bittencourt LR Tufik S Andersen ML . The effects of sleep deprivation and obstructive sleep apnea syndrome on male reproductive function: a multi-arm randomised trial. J Sleep Res. (2023) 32:e13664. doi: 10.1111/jsr.13664, PMID: 35670262

[96] Ding C Mao L Lu Y Wu S Ji W . Does obstructive sleep apnea-induced intermittent hypoxia increase the incidence of solitary pulmonary nodules, thyroid nodules, and other disorders? A retrospective study based on 750 cardiovascular disease patients. Sleep Breath. (2024) 28:1553–62. doi: 10.1007/s11325-024-03036-x, PMID: 38627339 PMC11303425

[97] Tan BKJ Tan NKW Teo YH Yap DWT Raghupathy J Gao EY . Association of obstructive sleep apnea with thyroid cancer incidence: a systematic review and meta-analysis. Eur Arch Otorhinolaryngol. (2022) 279:5407–14. doi: 10.1007/s00405-022-07457-w, PMID: 35708764

[98] Chen R Liang F Wang M Han P Lin P Zhang L . Impact of moderate-to-severe obstructive sleep apnea on aggressive clinicopathological features of papillary thyroid carcinoma. Sleep Med. (2022) 96:99–104. doi: 10.1016/j.sleep.2022.04.015, PMID: 35617717

[99] Choi JH Lee JY Lim YC Kim JK Do Han K Cho JH . Association between obstructive sleep apnea and thyroid cancer incidence: a national health insurance data study. Eur Arch Otorhinolaryngol. (2021) 278:4569–74. doi: 10.1007/s00405-021-06896-1, PMID: 34032908

[100] Bratel T Wennlund A Carlström K . Pituitary reactivity, androgens and catecholamines in obstructive sleep apnoea. Effects of continuous positive airway pressure treatment (CPAP). Respir Med. (1999) 93:1–7. doi: 10.1016/s0954-6111(99)90068-9, PMID: 10464840

[101] Mohammadi H Rezaei M Sharafkhaneh A Khazaie H Ghadami MR . Serum testosterone/cortisol ratio in people with obstructive sleep apnea. J Clin Lab Anal. (2020) 34:e23011. doi: 10.1002/jcla.23011, PMID: 31549459 PMC6977109

[102] Minami T Tachikawa R Matsumoto T Murase K Tanizawa K Inouchi M . Adrenal gland size in obstructive sleep apnea: Morphological assessment of hypothalamic pituitary adrenal axis activity. PloS One. (2019) 14:e0222592. doi: 10.1371/journal.pone.0222592, PMID: 31539392 PMC6754148

[103] Batura-Gabryel H Bromińska B Sawicka-Gutaj N Cyrańska-Chyrek E Kuźnar-Kamińska B Winiarska H . Does nesfatin-1 influence the hypothalamic-pituitary-gonadal axis in adult males with obstructive sleep apnoea? Sci Rep. (2019) 9:11289. doi: 10.1038/s41598-019-47061-3, PMID: 31383892 PMC6683188

[104] Luboshitzky R Lavie L Shen-Orr Z Lavie P . Pituitary-gonadal function in men with obstructive sleep apnea. The effect of continuous positive airways pressure treatment. Neuro Endocrinol Lett. (2003) 24:463–7., PMID: 15073577

[105] Luboshitzky R Aviv A Hefetz A Herer P Shen-Orr Z Lavie L . Decreased pituitary-gonadal secretion in men with obstructive sleep apnea. J Clin Endocrinol Metab. (2002) 87:3394–8. doi: 10.1210/jcem.87.7.8663, PMID: 12107256

[106] Bikov A Bailly S Anttalainen U Saaresranta T Basoglu OK Schiza S . Excessive daytime sleepiness, but not insomnia is associated with dyslipidaemia in patients with obstructive sleep apnoea participating in ESADA. J Sleep Res. (2025) 11:e70240. doi: 10.1111/jsr.70240, PMID: 41216973 PMC13193397

[107] Tamada D Otsuki M Kashine S Hirata A Onodera T Kitamura T . Obstructive sleep apnea syndrome causes a pseudo-Cushing’s state in Japanese obese patients with type 2 diabetes mellitus. Endocr J. (2013) 60:1289–94. doi: 10.1507/endocrj.ej13-0255, PMID: 24047562

[108] Parlapiano C Borgia MC Minni A Alessandri N Basal I Saponara M . Cortisol circadian rhythm and 24-hour Holter arterial pressure in OSAS patients. Endocr Res. (2005) 31:371–4. doi: 10.1080/07435800500456895, PMID: 16433255

[109] Hou N Ho JTF De Vries N De Lange J . Obstructive sleep apnea caused by acromegaly: Case report. Cranio. (2022) 40:451–3. doi: 10.1080/08869634.2020.1776530, PMID: 32485132

[110] Wang Y Zhou B Yue W Wang M Hu K . Glucose and lipid metabolism in non-diabetic, non-obese patients with obstructive sleep apnea: sex differences. Front Nutr. (2025) 12:1619371. doi: 10.3389/fnut.2025.1619371, PMID: 40909887 PMC12404915

[111] DunietzGL ChervinRD TaumanR ShaklaiS SankariA . Obstructive sleep apnea in women: associations with reproductive aging and screening challenges. Chest. (2025), S0012–3692(25)05125-6. doi: 10.1016/j.chest.2025.08.001, PMID: 40885537

[112] Tasali E Pamidi S Covassin N Somers VK . Obstructive sleep apnea and cardiometabolic disease: obesity, hypertension, and diabetes. Circ Res. (2025) 137:764–87. doi: 10.1161/CIRCRESAHA.125.325676, PMID: 40811500

[113] Gentile S Monda VM Guarino G Satta E Chiarello M Caccavale G . Obstructive sleep apnea and type 2 diabetes: an update. J Clin Med. (2025) 14:5574. doi: 10.3390/jcm14155574, PMID: 40807193 PMC12347911

[114] AlShawaf E Abukhalaf N AlSanae Y Al Khairi I AlSabagh AT Alonaizi M . Elevated IGFBP4 and cognitive impairment in a PTFE-induced mouse model of obstructive sleep apnea. Int J Mol Sci. (2025) 26:7423. doi: 10.3390/ijms26157423. Suwannakin A, Reutrakul S, Chirakalwasan N. Does glucose metabolism and its consequences depend on the phenotype of obstructive sleep apnea? Curr Opin Pulm Med. 2025 Aug 14. doi: 10.1097/MCP.0000000000001206., PMID: 40806552 PMC12348007

[115] Kurnia AD Thato R Tsai HT . Predicting factors of sleep quality among adults with type 2 diabetes mellitus: A systematic review. Diabetes Res Clin Pract. (2025) 227:112388. doi: 10.1016/j.diabres.2025.112388, PMID: 40774650

[116] Jia X Zou C Lu N Lu Q Xie C . Obstructive sleep apnea is ralated to metabolic dysfunction associated steatotic liver disease in type 2 diabetes mellitus. Sci Rep. (2025) 15:24627. doi: 10.1038/s41598-025-09985-x, PMID: 40634410 PMC12241494

[117] Passali D Bellussi LM Santantonio M Passali GC . A structured narrative review of the OSA-T2DM axis. J Clin Med. (2025) 14:4168. doi: 10.3390/jcm14124168, PMID: 40565914 PMC12194640

[118] Nithitsutthibuta K Sonsuwan N Uthaikhup S Kiatwattanacharoen S Kunritt J Pratanaphon S . Effects of obstructive sleep apnea on vascular structure and function in adults with obesity and type 2 diabetes: a comparative study. Cardiovasc Endocrinol Metab. (2025) 14:e00342. doi: 10.1097/XCE.000000000000034, PMID: 40756182 PMC12316327

[119] Insalaco G Braghiroli A Buzzi F Cappellano S Castelli A Castelnuovo A . Excessive daytime sleepiness and sex-related differences in the clinical presentation of obstructive sleep apnea in Italian patients. Sleep Med. (2025) 136:106819. doi: 10.1016/j.sleep.2025.106819, PMID: 41005183

[120] Henney AE Riley DR Anson M Heague M Hernadez G Alam U . Comparative efficacy of tirzepatide, liraglutide, and semaglutide in reduction of risk of major adverse cardiovascular events in patients with obstructive sleep apnea and type 2 diabetes: real-world evidence. Ann Am Thorac Soc. (2025) 22:1042–52. doi: 10.1513/AnnalsATS.202409-923OC, PMID: 40590655

[121] Nielsen S Nyvad J Grove EL Poulsen PL Laugesen E Christensen KL . Obstructive sleep apnea is associated with cardiac structural and functional alterations in patients with advanced diabetic kidney disease. Diabetes Res Clin Pract. (2025) 226:112225. doi: 10.1016/j.diabres.2025.112225, PMID: 40360122

[122] Quinaglia T Bakker JP Baltzis D Chan RH Manning WJ Wallace ML . Effect of continuous positive airway pressure on ventricular remodeling of patients with combined diabetes mellitus and obstructive sleep apnea: a cross-sectional study and follow-on randomized clinical trial. Int J Cardiol. (2025) 441:133788. doi: 10.1016/j.ijcard.2025.133788, PMID: 40825520 PMC12687071

[123] Phung L Duong K Obeid R . The utility of caffeine citrate as a neuroprotectant in the early life of premature newborns: a literature review of the effects on neurodevelopmental outcomes. Front Pediatr. (2025) 13:1682903. doi: 10.3389/fped.2025.1682903, PMID: 41488901 PMC12756483

[124] Xu C Owen JE Gislason T Benediktsdottir B Ye J Robinson SR . Limited microvascular remodelling occurs in the aged human hippocampus in obstructive sleep apnoea. Int J Mol Sci. (2025) 26:12040. doi: 10.3390/ijms262412040, PMID: 41465466 PMC12733236

[125] Iwhiwhu P Ben-Azu B Chijioke BS Ozah EO Friday FB Esuku DT . Combined maternal immune activation and prenatal intermittent hypoxic stress lead to developmental motor deficits in rats: a two-hit animal model for cerebral palsy across different age trajectories. Mol Neurobiol. (2025) 63:222. doi: 10.1007/s12035-025-05532-x, PMID: 41324817

[126] Liu L Zhang J Song S Li W Xiong W . Paraventricular nucleus neurons: important regulators of respiratory movement in mice with chronic intermittent hypoxia. Ann Med. (2025) 57:2588664. doi: 10.1080/07853890.2025.2588664, PMID: 41254959 PMC12632206

[127] Wang B Sun C Zhang R Gu A Zhao M Zhou X . MiR-106a-5p in extracellular vesicles derived from alveolar epithelial cells mediates cognitive dysfunction induced by chronic intermittent hypoxia in mice through MAPK signaling pathway. J Neuroinflammation. (2025) 22:291. doi: 10.1186/s12974-025-03628-8, PMID: 41388469 PMC12713293

[128] Zi Qian C Li Z Yi Ming L Teng H Lin Fan S Xiao Lei Z . Sex differences in endocrine, metabolic and psychological disturbance in obese patients with OSA. Biol Sex Differ. (2025) 16:48. doi: 10.1186/s13293-025-00730-7, PMID: 40598676 PMC12219881

[129] Shah SN Wright K Suh I Mahmoudi M Agrawal N . Enhanced detection of primary aldosteronism in hypertensive patients with obstructive sleep apnea using a novel diagnostic algorithm. Endocrine. (2025). doi: 10.1007/s12020-025-04322-8, PMID: 40526320

[130] Hundemer GL Imsirovic H Kendzerska T Vaidya A Leung AA Kline GA . Screening for primary aldosteronism among hypertensive adults with obstructive sleep apnea: A retrospective population-based study. Am J Hypertens. (2023) 36:363–71. doi: 10.1093/ajh/hpad022, PMID: 36827468 PMC10267649

